# The Allosteric Role of the AAA^+^ Domain of ChlD Protein from the Magnesium Chelatase of *Synechocystis* Species PCC 6803*

**DOI:** 10.1074/jbc.M113.477943

**Published:** 2013-08-12

**Authors:** Nathan B. P. Adams, James D. Reid

**Affiliations:** From the Department of Chemistry, The University of Sheffield, Sheffield S3 7HF, United Kingdom

**Keywords:** ATPases, Biosynthesis, Enzyme Catalysis, Mutagenesis Site-specific, Porphyrin

## Abstract

Magnesium chelatase is an AAA^+^ ATPase that catalyzes the first step in chlorophyll biosynthesis, the energetically unfavorable insertion of a magnesium ion into a porphyrin ring. This enzyme contains two AAA^+^ domains, one active in the ChlI protein and one inactive in the ChlD protein. Using a series of mutants in the AAA^+^ domain of ChlD, we show that this site is essential for magnesium chelation and allosterically regulates Mg^2+^ and MgATP^2−^ binding.

## Teaser

**Background:** Magnesium chelatase catalyzes the first essential step in chlorophyll biosynthesis.

**Results:** Mutations in the AAA^+^ domain of the magnesium chelatase ChlD subunit reduce but do not abolish catalytic activity.

**Conclusion:** ChlD is an allosteric regulator of magnesium chelatase.

**Significance:** These observations reveal an essential role for the ChlD protein in the first committed stage in chlorophyll biosynthesis.

## Introduction

In the first committed step in chlorophyll (Chl)^2^ biosynthesis, the enzyme magnesium chelatase (E.C. 6.6.1.1) inserts a magnesium ion into a porphyrin ring. This reaction is energetically unfavorable and only proceeds as it is coupled to an ATPase (1). In the minimal catalytic unit, the enzyme comprises three subunits (2–4). The largest of these subunits, ChlH, binds porphyrin and presumably contains the chelatase active site (5). The other two essential subunits, ChlI and ChlD, are both members of the AAA^+^ family of ATPases; but only one of these AAA^+^ subunits is an active ATPase (6).

The ATPase activity of ChlI is well characterized. This subunit acts an essential ATPase, providing free energy for the magnesium insertion reaction (6–8). The other AAA^+^ subunit, ChlD, has not been observed to catalyze ATP hydrolysis (6, 9). Although ChlD is absolutely required for catalysis, the role of ChlD in the chelation reaction is unknown.

ChlD contains three domains: a C-terminal integrin domain, a proline-rich linker domain, and an N-terminal AAA^+^ domain. The C-terminal integrin domain contains an essential MIDAS motif, proposed to be involved in Mg^2+^ binding (7, 10). The proline-rich linker is essential, but its role is unknown (11). We address here the significance of the AAA^+^ domain of ChlD and show that mutating conserved residues in the ATPase site of ChlD substantially reduces chelatase activity and specificity toward both MgATP^2−^ and Mg^2+^.

## EXPERIMENTAL PROCEDURES

All chemicals were obtained from Sigma unless otherwise stated. Porphyrins were obtained from Frontier Scientific (Logan, UT).

### 

#### 

##### Purification of Chelatase Subunits

The expression vectors pET9a-ChlI, pET9a-His_6_ChlD, and pET9a-His_6_ChlH were used to produce recombinant protein (12). ChlI and His_6_-ChlH were overexpressed in BL21(DE3) using the autoinducing medium ZYM-5052 (13) at 25 °C for 20 h. His_6_-ChlD was overexpressed in Rosetta2(DE3) pLysS, grown in enriched LB medium (16 g liter^−1^ tryptone, 10 g liter^−1^ yeast extract, 5 g liter^−1^ NaCl) at 37 °C and induced with 0.4 mm isopropyl 1-thio-β-d-galactopyranoside at an *A*_600_ of 0.8–1.0. The temperature was then lowered to 18 °C and cells harvested after 15 h. Proteins were then purified essentially as described previously (1, 12). Site-directed mutants were produced using the QuikChange method (Agilient, Cheshire, UK) and verified by sequencing (University of Sheffield, Core Genomics Facility).

##### Circular Dichroism Spectroscopy

Spectra were recorded with a JASCO-810 spectrometer (JASCO, Great Dunmow, UK). Protein (0.1 mg ml^−1^) was in 5 mm sodium phosphate buffer, 1 mm β-mercaptoethanol, pH 7.4. Spectra were recorded in a cuvette with a 0.2-cm path length. Spectra were recorded stepwise, from 250 to 190 nm (1-nm increments, 4 s/nm). Thermal stability was determined by monitoring the change in ellipticity of protein at 222 nm with temperature.

##### Co-purification of ChlID Complexes

Complex formation between His_6_-ChlD and ChlI was determined by mixing 2 μm ChlD and 2 μm ChlI (15 mm MgCl_2_, 50 mm Tricine, 0.3 m glycerol, pH 7.9) in the presence or absence of 5 mm ADP with 40 μl Ni(II) chelating Sepharose (GE Healthcare), at room temperature in a total volume of 200 μl. The resin was washed three times with buffer (as above), and 40 μl of 2× SDS-PAGE loading buffer was added directly to the resin and heated to 95 °C for 15 min. A 10-μl sample was analyzed by SDS-PAGE.

##### Assays of Enzyme Activity

Chelatase assays were performed at 34 °C, *I* 0.1, and pH 7.7 in 50 mm MOPS/KOH, 0.3 m glycerol, 1 mm DTT (1, 12). Free metal ion concentrations were controlled as described previously (1, 3, 8). Assays were initiated by the addition of enzyme to give a final concentration of 0.1 μm ChlD, 0.2 μm ChlI, and 0.4 μm ChlH. Substrate concentrations are given in the figure legends, but assays generally contained 15 mm MgCl_2_, 5 mm ATP, and 8 μm D_IX_. Deuteroporphyrin (D_IX_) solutions were prepared weekly in assay buffer and discarded if precipitated, magnesium deuteroporphyrin (MgD_IX_) was prepared on the day of use. ATP was determined spectrophotometrically, ϵ_260_ 15,400 m^−1^ cm^−1^ as were D_IX_ and MgD_IX_ (after the instantaneous conversion to D_IX_ in 0.1 m HCl), ϵ_398_ 433 000 m^−1^ cm^−1^ in 0.1 m HCl (14, 15).

Reactions were followed using a continuous fluorometric assay for MgD_IX_. Reactions (100 μl) were observed in a FLUOStar Optima plate-reader using a xenon flash lamp (BMG Labtech, Aylesbury, Buckshire, UK) with excitation through a 420-nm (bandpass 10 nm) filter and emission through a 580-nm (bandpass 10 nm) filter.

##### Kinetic Assays of Magnesium Chelatase Formation

Chelatase assays were performed as described above with 0.1 μm ChlD, 0.4 μm ChlH, and variable amounts of ChlI.

##### Data Analysis

Rates were estimated using the software supplied with the instrument (MARS Data Analysis Software Version 1.20 R2; BMG Labtech). Nonlinear regression was performed using Igor Pro 6.22A (Wavemetrics, Lake Oswego, OR). Kinetic parameters were determined by fitting Equation 1 or 2 to the data, and errors were determined from least squares analysis of the fits. To estimate *k*_cat_, the concentration of active enzyme was assumed to be the concentration of the ChlH subunit. In these equations, *K_m_* is the Michaelis constant, *K*_0.5_ is the substrate concentration that gives half-maximal velocity in the Hill equation, and *n* is the Hill parameter, an empirical measure of cooperativity.

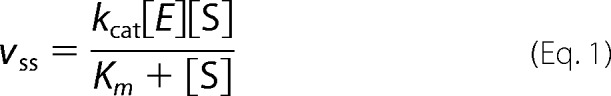


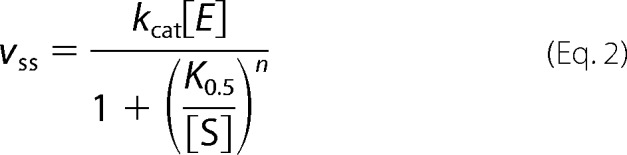


Assembly titrations were analyzed by fitting Equation 3 to the steady-state rates.

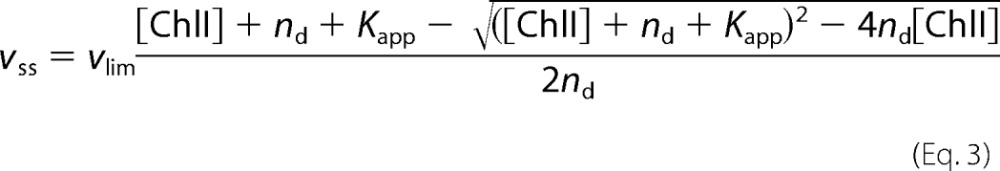


In Equation 3, *v*_ss_ is the observed steady-state rate, *v*_lim_ is the limiting steady-state rate reached at saturating ChlI, *n*_d_ is the concentration of binding sites for ChlI, *i.e.* [ChlD] × (number of sites/ChlD), and *K*_app_ is the apparent disassociation constant for the active ChlI-ChlD complex.

## RESULTS

### 

#### 

##### Choice and Characterization of Mutants

The ATP binding site is conserved across the AAA^+^ superfamily. Previous studies have shown that mutation of conserved residues in this site affects a range of steps in the ATP hydrolysis pathway, including nucleotide binding, nucleotide hydrolysis, and hydrolysis-dependent conformational changes (for review, see Refs. 16, 17). We selected residues for mutation that in other members of the superfamily are involved in all of these steps. The conserved lysine residue in Walker A motifs often binds nucleotide phosphates, and mutation of these residues frequently prevents or reduces nucleotide binding (18). In contrast, the conserved glutamate in the Walker B motif often activates water for nucleophilic attack on the terminal phosphate. Mutation of this residue frequently prevents nucleotide hydrolysis, but not nucleotide binding (18, 19). The Sensor II arginine often directly interacts with the β- and γ-phosphates of ATP, and mutations in this residue can prevent ATP binding or hydrolysis (17, 20). This arginine is found in the C-terminal helical domain, so if ChlD shows a domain rearrangement similar to that seen in the homologous BchI, this residue will be acting across the protomer interface (7). Arginine finger residues also act across protomer interfaces, and mutations in these residues often result in complete loss of activity (17, 20).

**FIGURE 1.**
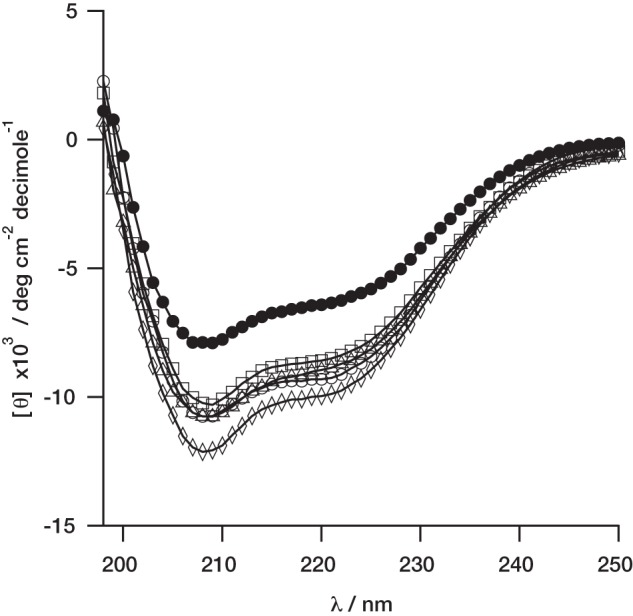
**CD spectra (mean residue ellipticity) of His_6_-ChlD variants (0. 1 mg ml^−1^) at 25 °C in 5 mm sodium phosphate buffer, 1 mm β-mercaptoethanol, pH 7.4.** *Traces* show wild type (○), K49A (□), E152Q (▵), R208A (♢), and R289A (●) His_6_-ChlD.

We have produced a series of mutants in the AAA^+^ domain of ChlD from *Synechocystis* sp. PCC 6803. These are K49A in the Walker A motif, E152Q in the Walker B motif, R208A removing the arginine finger and R289A removing the Sensor II arginine. All four mutant ChlD proteins can be purified by conventional methods. CD spectra for the wild type protein, K49A, and E152Q are essentially identical, whereas the R208A mutant shows a slightly increased helical content and the R289A mutant shows a reduced amount of secondary structure (Fig. 1). These CD spectra are all consistent with folded protein, although both arginine mutants (R208A and R289A) have some changes in secondary structure (Fig. 1).

**FIGURE 2.**
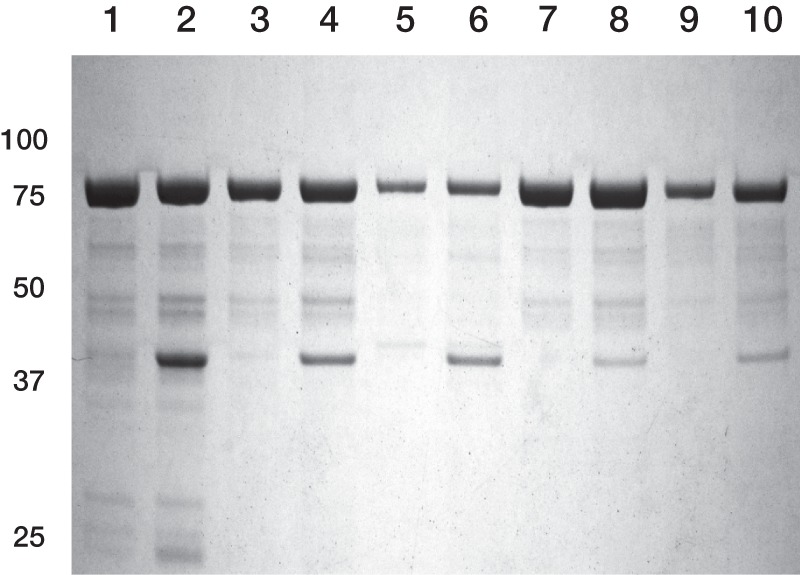
**ATP-dependent complex formation between wild type ChlI (2 μm) and mutant His_6_-ChlD (2 μm).** All samples contained ChlI and in addition, *lane 1*, wild type His_6_-ChlD and no ATP; *lane 2*, wild type His_6_-ChlD with ATP; *lane 3*, His_6_-ChlD (K49A) and no ATP; *lane 4*, His_6_-ChlD (K49A) with ATP; *lane 5*, His_6_-ChlD (E152Q) and no ATP; *lane 6*, His_6_-ChlD (E152Q) with ATP; *lane 7*, His_6_-ChlD (R208A) and no ATP; *lane 8*, His_6_-ChlD (R208A) with ATP; *lane 9*, His_6_-ChlD (R289A) and no ATP; *lane 10*, His_6_-ChlD (R289A) with ATP.

These residues are generally found at the interface between individual subunits in the oligomeric complex, and assembly of AAA^+^ complexes often depends on nucleotide binding (17). This suggests that mutating the ATPase sites could reduce the stability of the complexes. We determined whether these mutants form stable complexes with the ChlI protein. Intact complexes can be co-purified on a Ni^2+^ column (6). Control experiments demonstrate that ChlI does not bind these columns in the absence of nucleotide (Fig. 2, *odd numbered lanes*). All four of the mutant ChlD proteins discussed here bind to ChlI (Fig. 2). Co-purification does not allow us to quantify binding, but the R289A mutant appears to bind less ChlI than the other mutants or the wild type protein.

**FIGURE 3.**
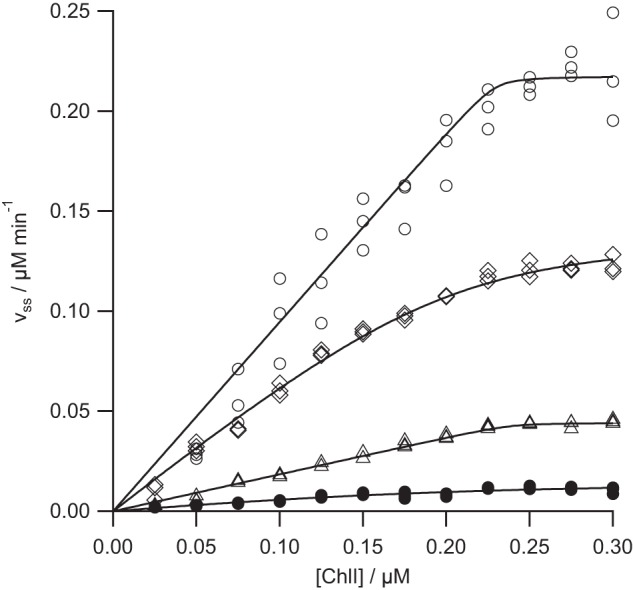
**Chelatase assembly titrations between wild type ChlI and wild type (○), E152Q (▵), R208A (♢), and R289A (●) His_6_-ChlD.** Assays contained 0.1 μm ChlD, 0.4 μm ChlH in 50 mm MOPS/KOH, 0.3 m glycerol, 1 mm DTT, 10 mm free Mg^2+^, *I* = 0.1 with KCl, 8 μm D_IX_, pH 7.7, 34 °C. The curves can be described by Equation 3 with the characterizing parameters: (wild type ChlD) *K*_app_ 0.17 ± 0.9 nm *n*_d_ 0.23 ± 0.01 μm and *v*_lim_ 0.22 ± 0.01 μm min^−1^; (E152Q) *K*_app_ 0.35 ± 7.3 nm *n*_d_ 0.24 ± 0.01 μm and *v*_lim_ 0.04 ± 0.01 μm min^−1^; (R208A) *K*_app_ 14 ± 11 nm *n*_d_ 0.20 ± 0.01 μm and *v*_lim_ 0.14 ± 0.01 μm min^−1^; (R289A) *K*_app_ 52 ± 155 nm *n*_d_ 0.19 ± 0.09 μm and *v*_lim_ 0.016 ± 0.009 μm min^−1^.

To quantify the binding interaction between ChlI and the ChlD mutants, we titrated ChlI against ChlD under assay conditions, monitoring the formation of porphyrin product. This assay directly evaluates the production of catalytically active complexes and allows us to disentangle the effects of mutation on complex formation (appearing in *K*_app_ and *n*_d_) and the activity of the complex (appearing in *v*_lim_). These titrations demonstrate that the arginine mutants have a reduced binding affinity, but that under our assay conditions (0.2 μm ChlI, 0.1 μm ChlD, 0.4 μm ChlH) the vast majority of ChlD is found in active complexes (Fig. 3).

Despite the low activity of the E152Q mutant, this species assembles into complexes very much like the wild type protein. The binding titration shows a characteristic tight-binding curve, with an estimated binding constant, *K*_app_, of 0.35 nm, much below the protein concentration in our assays (Fig. 3). The extent of complex formation seen with the E152Q mutant is essentially indistinguishable from the wild type protein with 99.6% of ChlD in the complex (extent of complex formation was calculated from Equation 3 and the parameters in Fig. 3).

The R208A mutant of ChlD will form an active chelatase complex, with the same optimal subunit ratio as the wild type enzyme. This mutant however shows weaker binding into the chelatase with a *K*_app_ of 14 nm (Fig. 3). As we are working with an excess of ChlI and at concentrations well above *K*_app_, the R208A mutant is overwhelmingly (approximately 89%) found in active chelatase complexes.

In contrast, the R289A mutant has a *K*_app_ of 52 nm, approaching the concentrations of protein used in these assays (Fig. 3). Nevertheless, at assay concentrations of ChlI and ChlD (0.2 and 0.1 μm, respectively), 71% of this mutant will be present in the active complex. This mutant shows <10% of the activity of the wild type (see below); this loss in activity cannot be attributed to impaired complex formation.

##### Steady-state Kinetic Analysis

**FIGURE 4.**
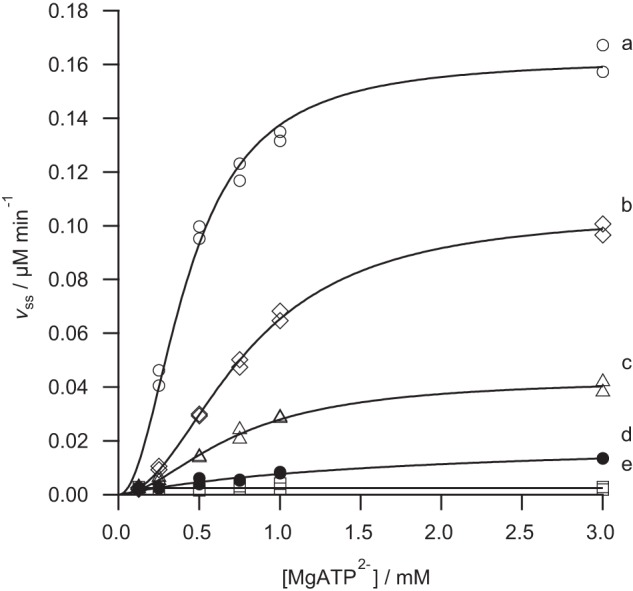
**MgATP^2−^ dependence of the steady-state chelation rate with wild type (*a*), R208A (*b*), E152Q (*c*), R289A (*d*), and K49A (*e*) variants of ChlD.** Assays contained 0.1 μm ChlD, 0.2 μm ChlI, 0.4 μm ChlH in 50 mm MOPS/KOH, 0.3 m glycerol, 1 mm DTT, 10 mm free Mg^2+^, *I* = 0.1 with KCl, 8 μm D_IX_, pH 7.7, 34 °C. The *lines* are described by Equation 2 with characterizing parameters *k*_cat_ 0.405 ± 0.014 min^−1^ *K*_0.5_ 0.43 ± 0.02 mm, *n* 2.0 ± 0.2 (*a*); *k*_cat_ 0.263 ± 0.005 min^−1^ *K*_0.5_ 0.78 ± 0.02 mm, *n* 2.0 ± 0.10 (*b*); *k*_cat_ 0.108 ± 0.004 min^−1^ *K*_0.5_ 0.72 ± 0.04 mm, *n* 1.8 ± 0.17 (*c*); and by the Michaelis-Menten equation *k*_cat_ 0.054 ± 0.005 min^−1^ *K_m_* 1.79 ± 0.32 mm (*d*). No activity was observed with the K49A variant.

Our series of mutants probe the role of the conserved AAA^+^ domain in ChlD. All of these mutant proteins are less active than the wild type enzyme (Fig. 4). The most active, the arginine finger mutant (R208A) shows just over 50% the activity of wild type enzyme, followed by the Walker B mutant (E152Q) and the Sensor II mutant (R289A). Metal ion chelation was undetectable with K49A, the Walker A mutant (Fig. 4). Clearly, the AAA^+^ site in ChlD is required for chelatase activity. We can gain further insight by examining the kinetic behavior of the mutant proteins in more detail.

**FIGURE 5.**
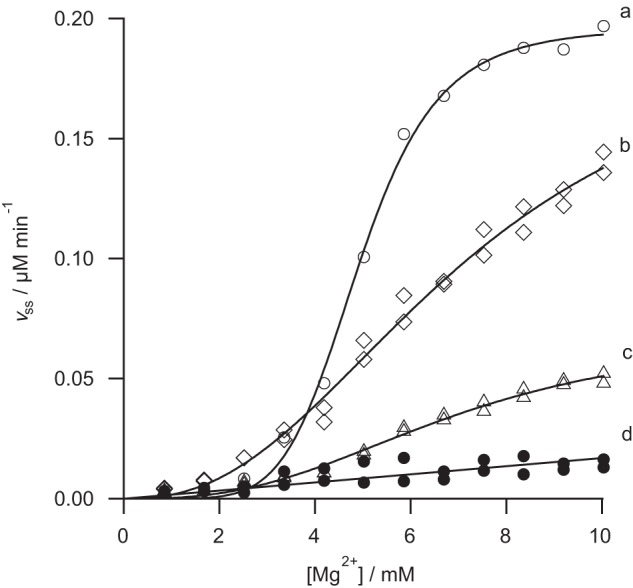
**Free magnesium dependence of the steady-state chelation rate with wild type (*a*), R208A (*b*), E152Q (*c*), and R289A (*d*) variants of ChlD.** Assays contained 0.1 μm ChlD, 0.2 μm ChlI, 0.4 μm ChlH in 50 mm MOPS/KOH, 0.3 m glycerol, 1 mm DTT, 5 mm ATP, *I* = 0.1 with KCl, 8 μm D_IX_, pH 7.7, 34 °C. The *lines* are described by Equation 2 with characterizing parameters *k*_cat_ 0.49 ± 0.01 min^−1^ *K*_0.5_ 4.93 ± 0.04 mm, *n* 6.0 ± 0.3 (*a*); *k*_cat_ 0.50 ± 0.06 min^−1^ *K*_0.5_ 7.18 ± 0.74 mm, *n* 2.4 ± 0.2 (*b*); *k*_cat_ 0.17 ± 0.02 min^−1^ *K*_0.5_ 6.97 ± 0.66 mm, *n* 2.7 ± 0.3 (*c*); and by a straight line equation *k*_cat_/*K_m_* 1.7 ± 1.0 min^−1^ mm^−1^ (*d*).

**FIGURE 6.**
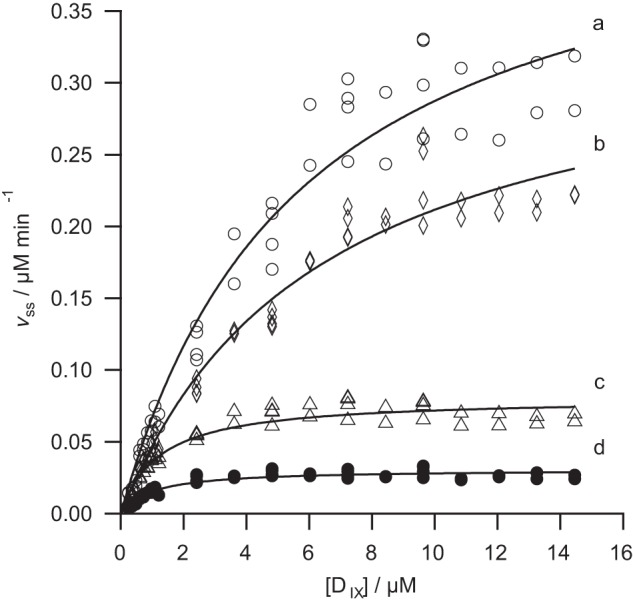
**Deuteroporphyrin dependence of the steady-state chelation rate with wild type (*a*), R208A (*b*), E152Q (*c*), and R289A (*d*) variants of ChlD.** Assays contained 0.1 μm ChlD, 0.2 μm ChlI, 0.4 μm ChlH in 50 mm MOPS/KOH, 0.3 m glycerol, 1 mm DTT, 5 mm ATP, *I* = 0.1 with KCl, pH 7.7, 34 °C. *Lines* are described by the Michaelis-Menten equation with the characterizing parameters *k*_cat_ 1.13 ± 0.06 min^−1^ *K_m_* 5.74 ± 0.77 μm (*a*); *k*_cat_ 0.86 min^−1^, *K_m_* 6.31 ± 0.75 μm (*b*); *k*_cat_ 0.20 ± 0.01 min^−1^ *K_m_* 1.23 ± 0.13 μm (*c*); and *k*_cat_ 0.08 ± 0.01 min^−1^ *K_m_* 0.99 ± 0.12 μm (*d*).

The R208A mutation removes the arginine finger. This protein retains substantial activity (*k*_cat_ >50% of wild type) and is the least impaired of all the mutants examined (Fig. 4). The effect of this mutation on substrate handling is likewise modest. Specificity for MgATP^2−^, *k*_cat_/*K*_0.5_, is approximately 35% of the wild type (Fig. 4). This is a significant reduction in *k*_cat_/*K*_0.5_, but not as much as would be expected if this residue were directly involved in nucleotide hydrolysis. Specificity constants for Mg^2+^ and porphyrin are approximately 70% of wild type (Figs. 5 and 6, respectively). This mutation has a small effect on specificity for Mg^2+^ and porphyrin.

The E152Q mutation in the Walker B motif also reduces the activity of magnesium chelatase. This mutation affects the specificity for MgATP^2−^ with *k*_cat_/*K*_0.5_ dropping to 16% of wild type (Fig. 4). Notably, this mutation also affects specificity toward Mg^2+^. The *k*_cat_/*K*_0.5_ for Mg^2+^ falls to approximately 30% of wild type, suggesting that this site affects metal-ion handling (Fig. 4). The effect on specificity for porphyrin is negligible because *k*_cat_/*K_m_* is essentially the same as the wild type enzyme (Fig. 6). In E152Q, both *k*_cat_ and *K_m_* for deuteroporphyrin are reduced to the same extent. These results fit well with the expected behavior of this mutant; in other members of the AAA^+^ superfamily analogous mutants bind but do not hydrolyze nucleotide (16).

The R289A mutation in the Sensor II motif has a profound effect on the catalytic properties of the chelatase. This mutant does not show a cooperative response to MgATP^2−^ and has at least an order of magnitude poorer specificity toward Mg^2+^. It is difficult to reach any mechanistic conclusion from a comparison of steady-state kinetic parameters of these cooperative and noncooperative enzymes as these parameters will arise from different sets of elementary rate constants. Nevertheless, the mutant *k*_cat_/*K_m_* of 0.3 m^−1^ s^−1^ is substantially lower than the wild type *k*_cat_/*K*_0.5_ of 15.7 m^−1^ s^−1^ (Fig. 4). Additionally, the magnesium specificity of the R289A mutant is substantially lower than the wild type (Fig. 5). This mutant shows a linear response to free magnesium, whereas all the other mutant proteins and the wild type enzyme show a cooperative, sigmoid, response. It is not possible to determine whether this mutant remains cooperative toward free magnesium. To define the shape of this curve, we would need to carry out assays at much higher concentrations of free magnesium (>50 mm ideally). Increasing the free magnesium concentration much above 10 mm will convert the substrate MgATP^2−^ into Mg_2_ATP. The observed linear response is consistent with a Michaelis-Menten response with *K_m_* ≫ 10 mm or with a Hill curve with *K*_0.5_ ≫ 10 mm.

The R289A mutant protein appears to have a lower specificity toward free porphyrin than the wild type enzyme (*k*_cat_/*K_m_* approximately 40% of wild type), but this mutant was not saturated with Mg^2+^. In the wild type enzyme, *k*_cat_/*K_m_* for porphyrin depends on the free Mg^2+^ concentration (1). So this apparent specificity constant for porphyrin only reflects the porphyrin specificity of this mutant at 10 mm Mg^2+^.

The K49A mutation in the Walker A motif inactivates magnesium chelatase. Mutations in Walker A motifs often produce proteins with poorer MgATP^2−^ binding (16). No activity is seen at 3 mm MgATP^2−^, by which point all the other proteins examined are saturated with nucleotide (Fig. 4). One difficulty in interpreting the behavior of the K49A mutant arises from the complete inactivity of this protein. It is not possible to verify magnesium chelatase complex formation by a kinetic assembly titration. As a result our evidence that this mutant can form intact complexes comes from the nucleotide-dependent co-purification of ChlI (Fig. 2). This co-purification demonstrates that the K49A mutant protein can form a detectable amount of ChlID complex, so the complete loss of activity implies that this complex is inactive.

## DISCUSSION

These results show that the AAA^+^ site in the D subunit of magnesium chelatase plays a significant role in the chelatase reaction. Mutations in this AAA^+^ site handicap chelation because the enzyme binds poorly to Mg^2+^ and MgATP^2−^.

The oligomerization of AAA^+^ ATPases is often sensitive to mutations in the ATPase site, with mutations in residues that act across the protomer interface (*e.g.* the arginine finger) or mutations that prevent nucleotide binding (*e.g.* Lys to Ala in the Walker A motif), preventing assembly (17). AAA^+^ proteins with two AAA^+^ domains in a protomer show a varied response to mutations in their ATPase sites; frequently one AAA^+^ domain is highly active, whereas the other is responsible for oligomerization (17). Whereas the magnesium chelatase subunits ChlI and ChlD each contain a single AAA^+^ domain, the combination of the two proteins is reminiscent of the twin domain members of the superfamily, and a similar division of labor has been proposed in magnesium chelatase (10).

Our activity titrations allow a simple, quantitative, assessment of the impact of these mutants on the formation of the magnesium chelatase complex. The wild type ChlD protein shows tight binding behavior, with an estimated *K*_app_ far below the concentration of protein used in the assays (Fig. 3). The E152Q mutation in the Walker B motif does not perturb complex formation to any detectable level (Fig. 3). This behavior is similar to that seen in many other members of the AAA^+^ superfamily, where Glu to Gln mutations in the Walker B motif generally allow nucleotide binding and nucleotide-dependent complex assembly (17).

Both arginine mutants show impaired assembly, although mutant complexes remain stable under our assay conditions; the R208A mutant has a *K*_app_ of 14 nm and the R289A mutant has a *K*_app_ of 52 nm (Fig. 3). Interestingly, mutation in the sensor two arginine of AAA^+^ ATPases rarely affects complex assembly (17). In the only available structure of an AAA^+^ subunit of magnesium chelatase, the C-terminal helical domain that contains the Sensor II arginine occupies an unusual rearranged position, adjacent to the next protomer ATPase site (7, 21). The reduction in binding affinity seen in our Sensor II arginine mutant is consistent with this residue acting across the protomer interface in the magnesium chelatase complex.

Most of these mutations have a modest effect on enzyme activity, suggesting that the AAA^+^ site on ChlD does not harbor an essential ATPase activity. If this site were an essential ATPase, all of these mutations would result in a substantial loss of chelatase activity. The modest reduction in activity observed with most of these mutants suggests that this site plays an allosteric role.

Magnesium chelatase with the K49A mutation in the Walker A motif of ChlD shows no detectable activity. In other AAA^+^ family members, this mutation is thought to impair nucleotide binding (16). This observation suggests that ChlD binds nucleotide. Taken together, the undetectable activity of the K49A mutant and the modest reduction in activity of the R208A mutant in the arginine finger and E152Q in the Walker B motif suggest that this AAA^+^ site is a nucleotide-binding allosteric regulator of magnesium chelatase.

The reduced *k*_cat_/*K*_0.5_^ATP^ of the arginine finger mutant (R208A) is consistent with the removal of an interaction between the guanidinium side chain and ATP γ-phosphate. It is not yet established which nucleotide species binds in the AAA^+^ site of ChlD, but these data suggest that ATP could bind in this site. Mutating the sensor two arginine produces an enzyme without a cooperative response to ATP. As the active ATPase sites are found in ChlI, this observation suggests that ChlD is involved in mediating communication between the individual ChlI subunits.

In the bacterial system, mutations in the sensor two position also cause substantial loss of activity. In *Rhodobacter capsulatus* the R194K mutation in BchD (*capsulatus* numbering) reduces activity to 25% of the wild type (10). This observation agrees with the data described here. The analogous mutation in *R. capsulatus* BchI (R298K) causes complete loss of chelatase activity (22). It would interesting to know whether Sensor II mutations in BchD from bacteriochlorophyll-producing systems also cause substantial changes in the response to free Mg^2+^ and MgATP^2−^.

The significance of individual domains in ChlD has been investigated in the tobacco enzyme (11). Gräfe *et al.* demonstrated that truncated mutants of ChlD, lacking the N-terminal AAA^+^ domain, retain near wild type activity. Curiously, the fusion protein where the N-terminal AAA^+^ domain of ChlD was replaced with that of ChlI was much less active than the wild type or the truncated protein. It is not immediately obvious how to reconcile our data with those of Gräfe *et al.* One speculative hypothesis is that nucleotide-free ChlD binds tightly to one of the other subunits, inhibiting the chelatase. On nucleotide binding, this interaction loosens and chelation is activated. In this model, truncating ChlD produces an unregulated constitutively active chelatase. Assessing this proposal will require further mechanistic study of truncated ChlD and, ideally, higher resolution structures of the ChlID complex. Current models of the ID complex are based on cryo-EM data and cannot provide information on the contents of nucleotide binding sites (21, 23). An isotope exchange study has demonstrated that an EPi complex is an intermediate on the ATP hydrolysis pathway (9). Still, these three-dimensional models of BchID show large conformational differences between the ATP, ADP, and the nonhydrolyzable analog AMPPNP bound forms, so they are consistent with this proposal (21).

Our data reveal that the AAA^+^ ATPase site in ChlD plays an important role in the reaction of magnesium chelatase. Although many questions remain to be answered, it is clear that the AAA^+^ site in ChlD is involved in the complex allosteric and cooperative response of magnesium chelatase to both Mg^2+^ and MgATP^2−^ and is involved in the communication pathway between the active ATPase sites in ChlI.

## Footnotes

2 The abbreviations used are:

Chl: chlorophyll
AMPPNP: 5′-adenylyl-β,γ-imidodiphosphate
D_IX_: deuteroporphyrin IX
MgD_IX_: magnesium deuteroporphyrin IX
Tricine: *N*-[2-hydroxy-1,1-bis(hydroxymethyl)ethyl]glycine.
